# The impact of in-hospital cardiac rehabilitation program on medication adherence and clinical outcomes in patients with acute myocardial infarction in the Lazio region of Italy

**DOI:** 10.1186/s12872-021-02261-6

**Published:** 2021-09-27

**Authors:** Salvatore Soldati, Mirko Di Martino, Alessandro Cesare Rosa, Danilo Fusco, Marina Davoli, Gian Francesco Mureddu

**Affiliations:** 1 Department of Epidemiology, Lazio Regional Health Service, Rome, Italy; 2 Department of Cardiovascular Diseases, S. Giovanni-Addolorata Hospital, Rome, Italy

**Keywords:** Cardiac rehabilitation, Acute myocardial infarction, Cardiovascular prevention, Epidemiology, Medication adherence, Long-term cardiovascular risk

## Abstract

**Background:**

Medication adherence is a recognized key factor of secondary cardiovascular disease prevention. Cardiac rehabilitation increases medication adherence and adherence to lifestyle changes. This study aimed to evaluate the impact of in-hospital cardiac rehabilitation (IH-CR) on medication adherence as well as other cardiovascular outcomes, following an acute myocardial infarction (AMI).

**Methods:**

This is a population-based study. Data were obtained from the Health Information Systems of the Lazio Region, Italy (5 million inhabitants). Hospitalized patients aged ≥ 18 years with an incident AMI in 2013–2015 were investigated. We divided the whole cohort into 4 groups of patients: ST-elevation AMI (STEMI) and non-ST-elevation AMI (NSTEMI) who underwent or not percutaneous coronary intervention (PCI) during the hospitalization. Primary outcome was medication adherence. Adherence to chronic poly-therapy, based on prescription claims for both 6- and 12-month follow-up, was defined as Medication Possession Ratio (MPR) ≥ 75% to at least 3 of the following medications: antiplatelets, β-blockers, ACEI/ARBs, statins. Secondary outcomes were all-cause mortality, hospital readmission for cardiovascular and cerebrovascular event (MACCE), and admission to the emergency department (ED) occurring within a 3-year follow-up period.

**Results:**

A total of 13.540 patients were enrolled. The median age was 67 years, 4.552 (34%) patients were female. Among the entire cohort, 1.101 (8%) patients attended IH-CR at 33 regional sites. Relevant differences were observed among the 4 groups previously identified (from 3 to 17%). A strong association between the IH-CR participation and medication adherence was observed among AMI patients who did not undergo PCI, for both 6- and 12-month follow-up. Moreover, NSTEMI-NO-PCI participants had lower risk of all-cause mortality (adjusted IRR 0.76; 95% CI 0.60–0.95), hospital readmission due to MACCE (IRR 0.78; 95% CI 0.65–0.94) and admission to the ED (IRR 0.80; 95% CI 0.70–0.91).

**Conclusions:**

Our findings highlight the benefits of IH-CR and support clinical guidelines that consider CR an integral part in the treatment of coronary artery disease. However, IH-CR participation was extremely low, suggesting the need to identify and correct the barriers to CR participation for this higher-risk group of patients.

**Supplementary Information:**

The online version contains supplementary material available at 10.1186/s12872-021-02261-6.

## Background

Cardiac rehabilitation (CR) is a multidisciplinary intervention, aimed at providing patients with cardiovascular disease with the optimum psychological and physical conditions to prevent their disease from progressing or potentially reversing its course [1]. Several studies have shown that participation in CR after acute myocardial infarction (AMI) is safe and effective to reduce morbidity, mortality, and hospital readmission rates by improving both risk factors’ control and adherence to treatments [2–5]. Despite these proven benefits, the referral of patients to CR is surprisingly low both in the hospital and in the ambulatory settings [6–8]. Given that AMI survivors have a higher incidence rate of readmission or major adverse cardiac events early after discharge as well as lifelong, they need secondary preventive care immediately after onset.

At the same time, medication adherence is a recognized key component of secondary cardiovascular disease prevention. With this regard, international guidelines recommended the combined use of cardioprotective drugs: platelet aggregation inhibitors (antiplatelets), β-blocking agents (β-blockers), angiotensin-converting enzyme inhibitors (ACEIs)/angiotensin receptor blockers (ARBs) as well as statins in combination with ezetimibe and PCSK9-inhibitors [9, 10]. Thus, adherence and persistence to chronic poly-therapy is a key factor in secondary prevention since it is associated with a lower risk of mortality and recurrent events [11–15].

CR programs offer a substantial contribution to achieving and maintaining lifestyle changes and medication adherence [16–19]. Hence, CR implementation is strongly recommended by clinical guidelines [20–23]. However, studies on the association between in-hospital CR intervention and adherence to evidence-based (EB) therapies in patients who suffering AMI are scarce in the Italian context. In the real-world, the absence of CR program may result in worse clinical outcomes at long-term follow-up.

The aim of our study was: (1) to evaluate the impact of the application of in-hospital CR program on adherence to chronic polytherapy following AMI and (2) to investigate the long-term effects of CR on three cardiovascular outcomes (all-cause mortality, hospital readmission for cardiovascular and cerebrovascular event, and admission to the emergency department) at three-year follow-up in the setting of the regional health service of Lazio region (Italy).

## Methods

### Data sources

We conducted a retrospective follow-up study using Italian administrative data. The data were extracted from the Lazio Regional Health Information Systems, which contain, for example, information on hospital admissions, in-hospital cardiac rehabilitation participation, mortality and drug claims. Details of all the individual information systems used in this research have been described in the Additional file 1: Data sources.

### Setting and study cohort

This is an observational study based on the population living in the Lazio region, Italy. Using data from the regional hospital information system (HIS), the study included a cohort of all patients discharged from hospitals between 1 January 2013 and 31 December 2015 with a diagnosis of AMI (see Additional file 2: Algorithm for selection of the cohort). In case of multiple hospital admissions, the first admission during the study period was defined as the index admission. ST-elevation AMI (STEMI) patients were identified using ICD-9-CM diagnosis codes 410.xx, excluding 410.7x (non-ST-elevation AMI) and 410.9x (acute AMI, not otherwise specified) in any diagnostic position. Instead, non-ST-elevation AMI (NSTEMI) patients were defined as a diagnosis codes 410.7x, excluding 410.9x. The validity of using this approach for defining AMI in administrative data has been previously documented [24]. Using ICD-9-CM procedure codes, we identified potentially AMI-related treatments provided during the hospital stay, including percutaneous coronary intervention (PCI, codes: 00.66, 36.01, 36.02, 36.05, 36.06, 36.07). Patients aged 18–100 years at discharge were screened for inclusion in the study. Only incident cases of AMI were included: patients with hospitalizations for AMI or related causes (i.e., percutaneous coronary intervention-PCI, bypass, ischemic heart disease, surgery of the heart and great vessels) in the 9 years before index admission were excluded from the analysis. Finally, patients who died or received an outpatient regimen for less than 30 days were excluded, to give all patients the chance to achieve clinical stability and to guarantee a minimum observation period of one month for consistently estimate adherence to polytherapy.

To investigate whether there might be subpopulations in which the effect of CR intervention is stronger, we divided the whole incident cohort into four different groups of AMI patients:

1. STEMI patients who underwent PCI (STEMI-PCI).
2. STEMI patients who did not receive PCI (STEMI-NO-PCI).
3. NSTEMI patients who underwent PCI (NSTEMI-PCI).
4. NSTEMI patients who did not receive PCI (NSTEMI-NO-PCI).

### In-hospital cardiac rehabilitation

In the Lazio region there are many CR institutes; during the recovery for an acute episode of AMI, the referral to a CR program was entirely at the discretion of the treating physician. In-hospital cardiac rehabilitation (IH-CR) consists of programs for prevention of deconditioning and recovery of daily activity in the acute phase, as well as supervised exercise therapy and patient education in the early recovery phase.

Patients candidates for access in IH-CR are those with in prolonged unstable conditions, undergoing evaluation for cardiac transplantation or verification of persistence of the indication, patients at high risk of new cardiovascular events and/or clinical instability, patients with a long in-hospital stay discharged after a prolonged stay in intensive care or intensive respiratory/cardiac care; patients with event-related complications, such as stroke, cognitive impairment, renal failure, pulmonary embolism, re-surgery, pleural or pericardial effusions requiring evacuation therapy, infections, complicated wounds, or those with presence or exacerbations of a severe comorbidities. IH-CR, by its multifactorial care structure based on a multidisciplinary team that involves not only the cardiologist but also the social, psychological, and behavioural dimensions of the disease is in a privileged position as regards the approach to the clinical complexity of multimorbidity and frailty.

The participation in IH-CR program was identified through a record linkage procedure between the HIS and the Regional Admission and Discharge Rehabilitation Information System (RAD-R). Therefore, patients who underwent in-hospital cardiac rehabilitation from specialized facilities were classified into the IH-CR group. Patients who did not participate in IH-CR program were classified into the non-IH-CR group. The IH-CR group was defined as patients who participated in IH-CR within 30 days from hospital discharge of the index episode of AMI.

### Outcomes

The primary outcome was adherence to EB therapies. Drug exposure information was collected from the regional registry of all drugs dispensed by public and private pharmacies. All drugs in this study were included in the patients’ health care plans and were equally available to all residents, in accordance with the universal health care coverage provided to residents of Italy. Information about prescriptions of antiplatelets (ATC: B01AC04, B01AC05, B01AC06, B01AC22), β-blockers (ATC: C07), ACEI/ARBs (ATC: C09), and statins (ATC: C10AA) were retrieved for all patients. Adherence to medication was measured through the medication possession ratio (MPR), calculated as the number of days of medication supplied during the follow-up based on defined daily doses (DDDs) divided by the number of calendar days in the follow-up. Adherence to chronic poly-therapy was defined as a MPR ≥ 0.75 for at least three of the four evidence-based drugs [15, 25].

Secondary outcomes were (1) all-cause mortality; (2) first hospital readmission due to major adverse cardiovascular and cerebrovascular events (MACCE), defined as a composite of death, recurrent myocardial infarction, ischemic stroke, and hospitalization for heart failure; (3) first admission to the emergency department (ED), occurring within a 3-year follow-up period.

### Follow-up

Adherence and persistence to polytherapy after the index event, were evaluated by analyzing prescription patterns during the 6- and 12-month following discharge from the index admission. Follow-up started 30 days after discharge from the index admission. The first 30 days after discharge were used to define exposure, to give all patients the opportunity to start a cardiological rehabilitation program. The end of individual follow-up for measuring drug adherence coincided either with the end of 6-month or 12-month follow-up, the date of death or with the date of all-cause hospitalization whichever came first. Regarding to the secondary outcomes, patients were followed from the 30^th^ day after hospital discharge (the first 30 days after discharge were used to define exposure) to the occurrence of individual events, censoring at the date of non-cardiovascular mortality or the end of 3-year follow-up for MACCE admissions, and censoring at the date of all-cause mortality or the end of 3-year follow-up for admissions to the emergency department.

### Statistical analysis

Data are presented as frequencies and percentages for categorical variables and median value (quartile 1–quartile 3) for continuous variables. The impact of IH-CR on adherence to chronic poly-therapy following an AMI was analysed using logistic regression models. Potential confounders were selected based on a priori knowledge [26–28], including the following: gender, age and 18 relevant comorbidities retrieved from the hospital records for both the index admission and the two previous years (see Additional file 3: Selection of comorbidities from hospital discharge records). Odds ratios (OR) with the corresponding 95% confidence intervals (95% CI) and *p *values were computed. We tested the potential interaction between IH-CR participation and sub-cohorts (four distinct groups) of AMI patients by including a cross-product term to each model. The statistical significance of the coefficients for each cross-product term was evaluated using the likelihood-ratio test.

To determine the impact of IH-CR participation on all-cause mortality, hospital readmission due to MACCE, and admission to ED, we used a Poisson regression models (counts divided by person-time). The three secondary outcomes were analyzed individually. All models included the interaction between IH-CR participation and the patient’s subgroup membership and were adjusted for the potential confounders listed in the Additional file 3: Selection of comorbidities from hospital discharge records. Interaction terms were considered statistically significant at *P* values < 0.10.

Statistical analyses were carried out using Stata software, version 15 (StataCorp.2015. Stata Statistical Software: Release 15. College Station, TX: Stata Corp LP).

## Results

### Patients’ characteristics and IH-CR participation

**Fig. 1:**
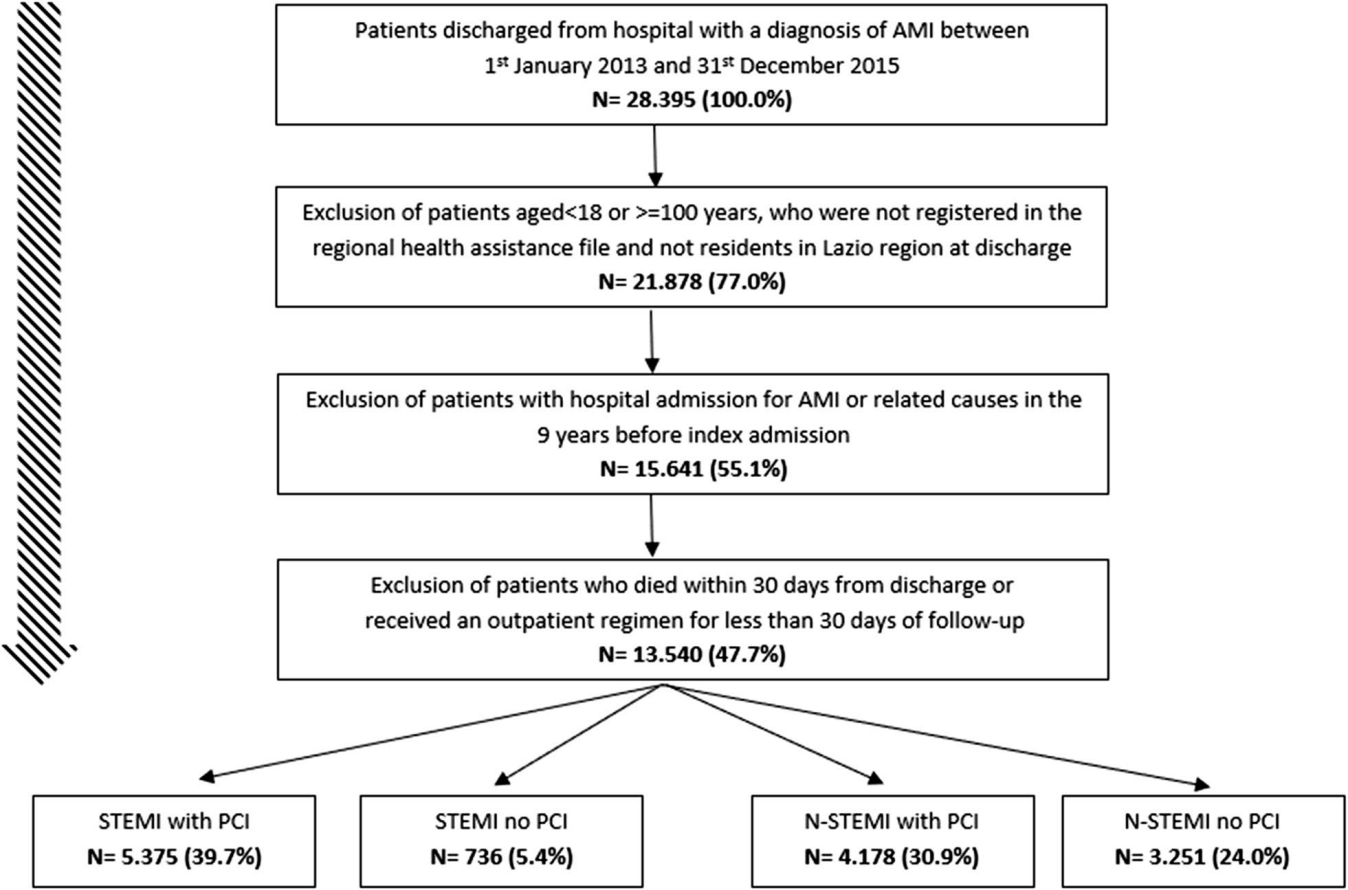
Cohort selection. Exclusion criteria flow chart

The flow chart in Fig. 1 shows the selection process of the study cohort. Of the 28.395 patients discharged from hospital with a first diagnosis of AMI between January 1st 2013 and December 31th 2015, 13.540 (48%) met the inclusion criteria and were enrolled in the present study. Among the entire study population, 1.101 (8.0%) patients began IH-CR at 33 regional sites, during the 30 days following hospital discharge from the index admission. However, the proportion of patients that have participated in IH-CR program strongly changes according to the subgroup of AMI patients identified. Participation in IH-CR ranged from 3 to 17%. Table 1 lists the main characteristics of AMI subpopulations, grouped by IH-CR participation status.

**Table 1:** Clinical and procedural characteristics of AMI patients grouped by IH-CR status

	AMI overall (N = 13.540)		STEMI-PCI (N = 5.375)		STEMI-NO-PCI (N = 736)		NSTEMI-PCI (N = 4.178)		NSTEMI-NO-PCI (N = 3.251)	
	IH-CR		IH-CR		IH-CR		IH-CR		IH-CR	
	No	Yes	No	Yes	No	Yes	No	Yes	No	Yes
Patients, n (%)	12.439 (91)	1.101 (8)	5.084 (95)	291 (5)	612 (83)	124 (17)	4.038 (97)	140 (3)	2.705 (83)	546 (17)
Age, median (IQR) years	67 (57;77)	69 (60;77)	63 (55;72)	66 (57;76)	77 (64;85)	68 (61;78)	67 (58;76)	73 (62;80)	75 (63;84)	71 (61;77)
Male sex	66.00	70.66	76.95	70.10	41.83	76.61	72.01	67.14	41.92	70.52
Length of IH-CR stay, median (IQR) days	–	22 (17;26)	–	20 (15;25)	–	22 (18;26)	–	20 (16;25)	–	22 (18;26)
Malignant neoplasm	4.16	5.36	3.34	5.15	6.54	6.45	3.84	4.29	5.66	5.49
Diabetes	3.54	5.63	2.34	3.78	5.07	5.65	3.81	4.29	5.03	6.96
Disorders of lipoid metabolism/obesity	4.35	5.18	4.21	3.44	3.59	2.42	4.95	4.29	3.88	6.96
Haematological diseases	3.70	4.27	1.81	4.12	8.82	2.42	2.77	2.86	7.47	5.13
Hypertension	6.29	7.99	3.86	5.15	9.97	7.26	6.51	7.86	9.69	9.71
Heart failure	11.65	25.79	7.45	29.21	22.06	26.61	8.94	24.29	21.22	24.18
Other cardiac diseases	4.28	7.90	1.87	4.47	8.99	13.71	3.05	6.43	9.57	8.79
Conduction disorders/cardiac arrhythmias	20.95	26.07	19.47	31.96	22.55	21.77	16.72	27.14	29.69	23.63
Cerebrovascular disease	5.07	12.62	3.17	6.53	8.66	12.10	4.8	10.71	8.24	16.48
Diseases of arteries, arterioles, and capillaries	3.75	5.36	3.36	3.78	3.10	4.84	4.38	3.57	3.66	6.78
Chronic obstructive pulmonary disease (COPD)	6.74	7.99	4.31	4.81	10.29	7.26	6.19	10.00	11.35	9.34
Chronic nephropathies	7.38	8.45	4.41	5.84	10.95	5.65	6.44	6.43	13.57	10.99
Chronic liver, pancreas, and digestive diseases	0.88	0.54	0.77	0.69	1.47	0.81	0.82	-	1.04	0.55
Cerebrovascular revascularization	0.42	1.36	0.26	–	0.33	–	0.57	–	0.52	2.75
Other operations on heart and pericardium	1.20	12.99	1.42	11.34	1.31	16.13	0.72	6.43	1.48	14.84
Other operations on vessels	1.48	3.18	0.98	3.44	1.63	4.03	1.31	3.57	2.62	2.75

AMI, acute myocardial infarction; IH-CR, in-hospital cardiac rehabilitation

IH-CR participants were more frequently male, slightly older, and had a greater burden of comorbidities and interventional procedures. In fact, the participants were more likely to have a history of heart failure, conduction disorders or arrhythmias or other cardiac diseases, were more likely to be seen with cerebrovascular diseases or more likely to be treated with other operations on heart and pericardium, compared with those who did not participate in IH-CR program. However, regarding these percentages, more or less evident differences can be observed across the 4 subgroups of patients. In addition, the median length of IH-CR stay ranged between 20 to 22 days.

### Impact of IH-CR on adherence to chronic polytherapy

The adherence to EB medications by AMI subpopulations and IH-CR participation status is reported in Table 2. Overall, 44% and 51% of the patients were deemed adherent to polytherapy during the 6-and 12-month follow-up. Subgroup analyses revealed that STEMI-PCI patients were characterised by the highest adherence, for both 6-and 12-month follow-up (52% and 60%, respectively), followed by NSTEMI-PCI patients (49% and 56%). The adherence to therapies decreased markedly, for both STEMI and NSTEMI, considering those patients who did not undergo PCI: (31% and 35% for STEMI), (29% and 33% for NSTEMI patients).

**Table 2:** Adherence to chronic poly-therapy at 6- and 12-month follow-up by AMI groups

AMI patients	Adherence at 6-month follow-up (%) (MPR ≥ 75% at least 3 of 4 E-B drugs)			Adherence at 12-month follow-up (%) (MPR ≥ 75% at least 3 of 4 E-B drugs)		
	Overall	IH-CR		Overall	IH-CR	
		No	Yes		No	Yes
STEMI-PCI	51.91	52.20	46.56	59.92	60.31	52.67
STEMI-NO-PCI	30.87	29.11	39.64	35.24	33.27	45.05
NSTEMI-PCI	48.82	48.96	44.62	55.91	56.25	46.15
NSTEMI-NO-PCI	29.38	27.22	40.24	33.48	30.55	48.21

AMI, acute myocardial infarction; IH-CR, in-hospital cardiac rehabilitation

**Fig. 2:**
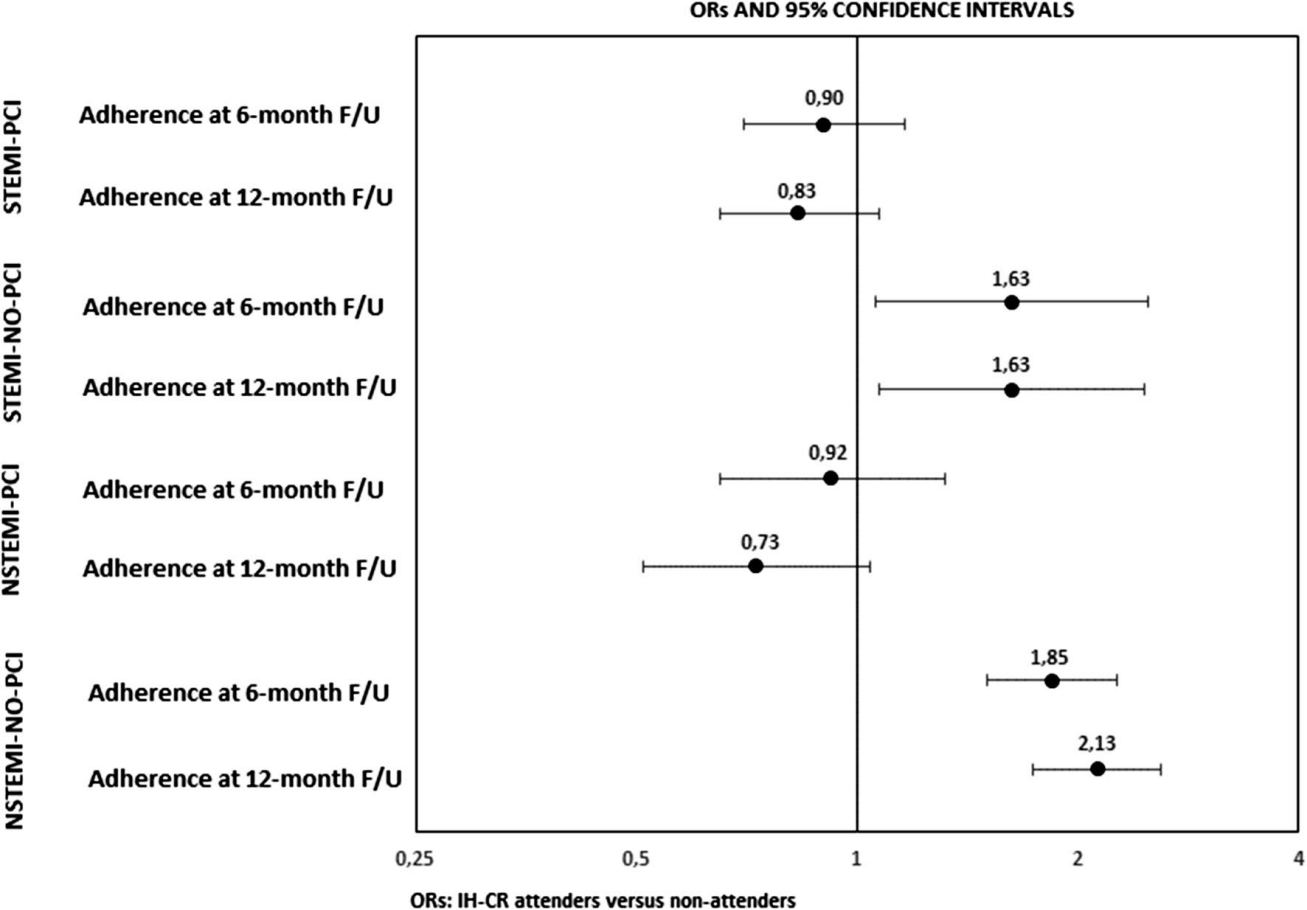
The effect of IH-CR on adherence to poly-therapy at 6-and 12-month follow-up, by AMI groups

Using the logistic models with term-interaction between IH-CR participation and 4 subpopulations of AMI patients, the impact of IH-CR participation on adherence to chronic poly-therapy was determined. The “global” interaction term was statistically significant (*p *value < 0.001), for both 6- and 12-month follow-up. As visualized in Fig. 2, the effect of cardiac rehabilitation on adherence to medications differed considerably between subpopulation groups. IH-CR participation was associated with a statistically significant increase in adherence to chronic poly-therapy at 6-month (OR 1.63; 95% CI 1.06–2.50; *p *value: 0.02) for STEMI-NO-PCI patients, and this improvement was also maintained at 12-month follow-up assessment (OR 1.63; 95% CI 1.07–2.47; *p *value: 0.02). A similar but even stronger effect was observed for NSTEMI-NO-PCI patients, who attended the IH-CR program compared with non-attenders at 6-month (OR 1.85; 95% CI 1.51–2.27; *p *value < 0.001) and at 12-month follow-up assessment (OR 2.13; 95% CI 1.74–2.60; *p *value: < 0.001). In contrast, the effect of IH-CR intervention on adherence was not observed in STEMI-PCI group (OR 0.90; 95% CI 0.70–1.16; *p *value: 0.427), (OR 0.83; 95% CI 0.65–1.07; *p *value: 0.157), at 6-and 12-month follow-up, respectively. A similar non-significant trend was observed among NSTEMI-PCI patients.

### Impact of IH-CR on the occurrence of secondary outcomes

**Fig. 3:**
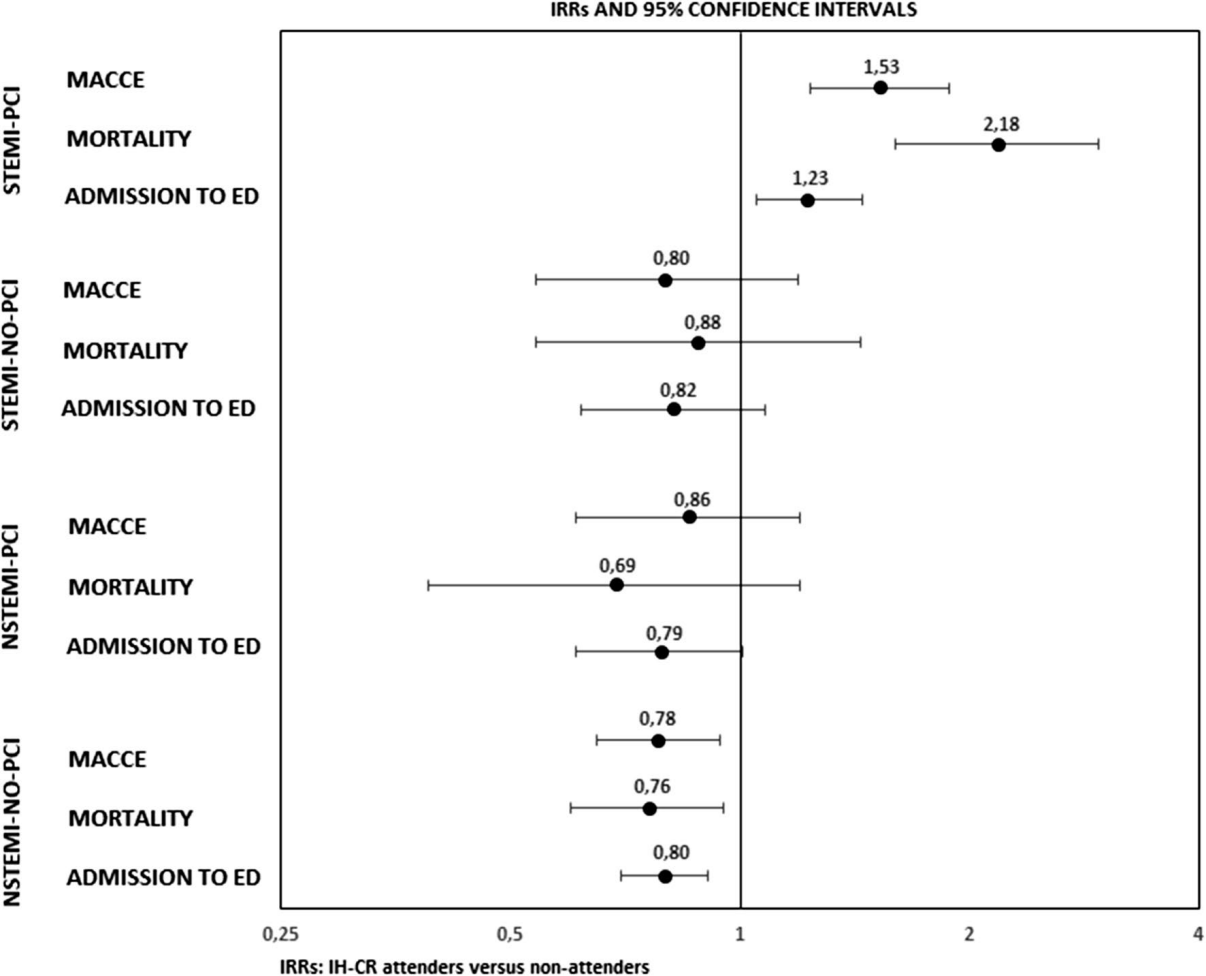
The effect of IH-CR on incidence of secondary outcomes at 3-year follow-up, by AMI groups

During the 3-year follow-up, there were 1.635 all-cause deaths in our population cohort. In addition, admission to emergency department was noted in 6.928 patients, while hospital readmission due to MACCE was noted in 3.302 patients. The 3-year follow-up rates of these outcomes were 12%, 51%, and 24%, respectively. As regards the Poisson regression models, the global interaction term between IH-CR participation and the patient’s subgroup membership was statistically significant (*p *value < 0.001) for each of the three secondary outcomes. As shown in Fig. 3, the participation in cardiac rehabilitation would appear to have a “protective effect” on the occurrence of the adverse secondary outcomes, for three out of the four sub-cohorts identified. Specifically, for the NSTEMI-NO-PCI group, this protective effect of IH-CR was statistically significant for all three adverse outcomes. Incidence rates of all-cause mortality, hospital readmission due to MACCE and admission to ED were significantly lower in the IH-CR participants than in the non-participants. The relative risks were 0.76 (95% CI 0.60–0.95), 0.78 (95% CI 0.65–0.94), and 0.80 (95% CI 0.70–0.91), respectively.

Surprisingly, in the first group of AMI patients (STEMI-PCI), the effect of IH-CR participation on the occurrence of secondary outcomes switched from protective factor to risk factor. We found for this specific group, significantly higher incidence rates for all secondary outcomes, among patients who participated at IH-CR compared with non-participants.

## Discussion

This study provides novel ‘real world’ data on the referral and impact of in-hospital cardiac rehabilitation programs in patients suffering AMI. The study involved more than 13.000 patients admitted to hospitals in the Lazio region of Italy between 2013 and 2015.

The study results showed that IH-CR participation rates are alarmingly low, ranging from 3 to 17% in relation to the groups of the AMI population identified. The highest IH-CR referral rate were observed for STEMI and NSTEMI patients who did not receive PCI during the index hospitalization. Overall, we found that only 1.101 patients (8%) started cardiac rehabilitation program within 30 days from the hospital discharge following an AMI.

As evidenced by the results of this study, IH-CR referral rates have been very low in comparison with other E-B treatments. Previous investigations from several states reported an average referral rate of 30% in Canada, the USA, and the UK, and a little higher at 50% in the rest of Europe [29]. Differences in the organization of health care services and delivery systems between countries and across hospitals may explain, most probably, this variability. Moreover, there are also differences in referral rates between European countries, but little is known, in literature, about the real motivations for underuse of cardiac rehabilitation in different EU countries and hospitals [10, 30]. However, these current low rates of participations in IH-CR programs are wholly inadequate and deprive a large proportion of patients with cardiovascular disease of a safe and effective intervention in reducing disease-related disability, as well as hospital readmission and long-term mortality. There are several reasons for this inadequate use of CR. First of all, the lack of a hospital system that automatically associates the rehabilitation program with each eligible patient, without the intermediation of a physician or health care staff. In fact, previous studies suggested that the most important predictor, is exactly the lack of CR referral at hospital discharge [31].

Sometimes, it may be that patients do not receive enough information and encouragement to participate in CR by their physician or other health professionals. However, physicians certainly need to take into consideration many factors when giving a CR referral: a patient could be too old or frail to benefit from CR, or conversely, too healthy and may not need this intervention. Last but not least, CR regimes are not standardized across countries regarding duration (3/6 weeks or 12/16 weeks), setting (in-hospital or ambulatory), and main goal of therapy (exercised based or comprehensive recommending). In Italy, there are no top-down standards or guidelines regarding the scope of services in IH-CR. The gold standard of care refers to the guidelines drawn up from the scientific societies. The need for a national clinical register in Italy, which supports the standardization of interventions has been repeatedly advanced by the scientific community [32]. The procedures, the personnel, and modalities necessary for carrying out the activities that can be defined as "rehabilitation intervention", have been also specified. However, at least in the Lazio region there are no steering documents that make the standard of care in CR mandatory [33]. Moreover, this work increases knowledge and awareness that, even in the most advanced interventional cardiology contexts, where networks hub and spoke for acute myocardial infarction have already been developed for two decades, post-acute care is still very lacking. In fact, consistent with the results of other investigations [26–28, 34] in the present study we found that evidence-based pharmacological therapies for secondary prevention after AMI were under-used (overall, 44% and 51% of the patients were deemed adherent to poly-therapy at 6-and 12-month, respectively).

Our findings showed a differential impact of cardiac rehabilitation program on polytherapy adherence both at 6-and 12-month follow-up assessment. The magnitude of this impact depending on the different groups of AMI patients considered, which are related to IH-CR participation. Principally, there would seem to be a strong different impact between patients who underwent or not PCI, regardless of the AMI diagnosis, during the index hospitalization. In fact, during the 6-month follow-up, after adjusting for potential confounders, STEMI-NO-PCI and NSTEMI-NO-PCI patients who started IH-CR program were, 63% and 85% respectively, more likely to be adherent as compared with non-participants. Interestingly, polytherapy adherence was maintained for STEMI-NO-PCI participant patients (63%), and even increased for NSTEMI-NO-PCI patients at 12-month follow-up, suggesting that patients who could not undergo revascularization by coronary angioplasty may have been more often referred to IH-CR than those who received coronary angioplasty. Likewise, the rehabilitation intervention results in a greater adherence to drug therapy. Among NSTEMI-NO-PCI patients who participated in IH-CR were more than twice as likely to be adherent to E-B drugs (OR 2.13; 95% CI 1.74–2.60; *p *value: < 0.001). Of note, estimates were adjusted for all variables identified as potential confounders such as age, gender, and 18 comorbidities listed in the Additional file 3: Selection of comorbidities from hospital discharge records. By contrast, the effect of IH-CR intervention on adherence was not observed in the other two subgroups of AMI patients (STEMI and NSTEMI) who underwent PCI during the index event. This result may be partially due to the fact that AMI patients who underwent a primary percutaneous coronary intervention have already been inserted in specific care pathways. In fact, these two groups of patients, regardless of IH-CR participation status, showed the highest adherence, for both 6-and 12-month follow-up (52% and 60% for STEMI; 49% and 56% for NSTEMI patients). Therefore, the beneficial effect of IH-CR treatment on medication adherence could be attenuated by the fact that AMI patients who underwent revascularization had been more carefully monitored and made aware of the long-term benefits generated by a continuous and persistent drug treatment.

The evidence of greater effect of IH-CR on the adherence to long-term evidence-based drugs in more complex groups, i.e., in patients who have had an acute coronary event but not revascularized, reinforces the concept that rehabilitation intervention is extremely relevant from a prognostic point of view. Non-revascularized patients with multiple comorbidities are recognized as those with the highest residual cardiovascular risk. Numerous clinical, epidemiological and intervention studies confirm this finding. In Italy, data from the National Outcomes Program (NOP) of 2016 show a reduction in mortality at 30 days from admission for acute myocardial infarction (AMI), from 10.4% in 2010 to 9.0% in 2015. An Italian retrospective study based on the administrative database of hospital discharge forms (HDF) examined over one million patients admitted for AMI from 2001 to 2011 in all Italian hospitals, evaluating the mortality and hospital readmission rates at 30 days and at 1 year and re-hospitalization for all causes. While the mortality rate at the index event declined the rate of fatal readmission at 1 year increased from 4.75 to 5.28% (*p* = 0.0019) [35]. It thus emerges that, while the in-hospital management of ACS has shown great progress in terms of its diagnostic-therapeutic efficacy, post-discharge care has not had a positive impact on the post-discharge prognosis of ACS. This is in part attributable to the inadequacy and poor application of appropriate cardiac care pathways for the post-discharge period, based on different care needs modelled according to the individual patient’s level of risk. A high residual risk of MACCE recurrence can be detected both by clinical factors, such as diabetes mellitus, renal failure, peripheral artery disease, a history of angina or previous AMI, and by anatomical/surgical factors, such as the presence of multivessel disease, especially if treated with incomplete revascularization, or no revascularization at all. Elderly age is an associated high-risk factor included in the above comorbidities. A recent Italian study, based on HDF administrative data, analysed the risk of thrombosis in patients admitted in the years 2009 and 2010. At multivariate analysis, most of the factors defining high thrombotic risk were independent predictors of 5-year mortality. The effect of "thrombotic risk" on mortality resulted to be time-dependent with a hazard ratio (HR) that strongly increased in the first two years of follow-up, then slowly levelled out in the following years to reach a plateau around the 5th year. This finding confirms not only the importance of targeting intensive prevention therapeutic strategies in the period immediately following ACS but points to the need for long-term secondary prevention programs in subgroups of high-risk patients [13]. Intervention studies have confirmed this epidemiological trend as regards dual antiplatelet therapy [36–38], LDL target [39, 40] and even low-dose anticoagulation in the more recent COMPASS trial (Cardiovascular Outcomes for People Using Anticoagulation Strategies) [41]. In this context, therefore, adherence to evidence-based therapy, as well as implementation of new secondary prevention pharmacological strategies in patients at very high residual risk plays a fundamental role. Unfortunately, despite the evidence, as also evidenced by the results of this research, the adherence to pharmacological therapies after an acute event is still far from optimal.

Moreover, the present study investigated the long-term effects of IH-CR intervention on all-cause mortality, hospital readmission due to MACCE, and admission to ED at 3-year follow-up. As previously mentioned, the participation in IH-CR had a “protective effect” on the occurrence of these three adverse secondary outcomes, for three out of the four subpopulations identified. However, this protective effect was statistically significant only for NSTEMI-NO-PCI patients. The study results showed that IH-CR participation, was associated with 24% of risk reduction of mortality (IRR 0.76; 95% CI 0.60–0.95; *p *value: 0.021), 22% of MACCE readmission (IRR 0.78; 95% CI 0.65–0.94; *p *value: 0.011), and 20% of ED admission (IRR 0.80; 95% CI 0.70–0.91; *p *value: 0.001). Much of this effect is probably due to the increase in adherence to evidence-based therapies. Our findings are consistent with the results of other investigations that reported a relationship between CR participation and risk reduction for AMI patients [42–44]. In addition, meta-analyses suggested that CR participation can reduce all-cause mortality by 15–28% for AMI patients [45, 46], confirming our results. We should be pointed out how NSTEMI-NO-PCI patients had the highest percentage of patients who participated in IH-CR intervention (17%) compared to the other AMI subgroups. Moreover, in absolute terms, patients with a diagnosis of NSTEMI who did not undergo PCI, represented the 50% of all AMI patients that have participated in IH-CR program in this research.

Rather, we found that all these secondary outcomes tended to be higher in the IH-CR group for STEMI-PCI patients. However, based on the factors showed in Table 1, it appears evident a different severity of patient’s condition who participated or not to IH-CR program in this specific subgroup of population. In fact, IH-CR participants were more likely to have a history of heart failure (29% vs. 7%), conduction disorders or arrhythmias (32% vs. 19%), and were more likely to be treated with other operations on heart and pericardium (11% vs. 1%), compared with those who did not participate in IH-CR program. Consequently, all-cause mortality tended to be higher in the IH-CR group (17% vs. 6%).

Notable, the median follow-up time to death for patients who participated in IH-CR was 384 days (IQR 161–688) versus 603 days (IQR 301–822) for those who did not participate. For the above-mentioned reasons, it is reasonable to expect a strong disparity in the characteristics of patients who did or did not participate in IH-CR even for all variables that cannot be measured. Although we evaluated all available potential factors to adjust patient deviation, we could not adjust for imbalance in unmeasured confounders. Therefore, most likely the lack of more detailed data (e.g., ejection fraction, history of smoking, BMI, number of sessions completed, exercise capacity, vital sign, functional status, frailty, or social risk factors) has caused unmeasured confounding resulting in higher incidence rates for secondary outcomes, among STEMI-PCI patients who participated in cardiac rehabilitation compared with non-participants.

### Strengths and limitations of the study

A strength of the study is the possibility to integrate different Health Information Systems of the Lazio region of Italy, in order to achieve a well-defined (e.g., chronological, demographical, clinical) healthcare-related patient profile and to involve a large number of patients. The robustness of the applied statistical methodologies, a population study design and a long-term follow-up are the major strengths. A weakness of this study is that the results derived from a single region of Italy, so may not be generalizable to other contexts due to differences in healthcare policies. Moreover, a misclassification of drug utilization may have occurred because in our database the prescribed daily doses were not known, and the defined daily doses were used as the dosage assumption to measure adherence to polytherapy [47, 48]. In addition, we only used data from RAD-R for the definition of study’s exposure (participation in cardiac rehabilitation). This information system collects data about patients referred to CR specialized facilities from hospital acute care centers and may not reflect the experience of other cardiac rehabilitation centers. Therefore, the IH-CR participation proportion may have underestimated. Finally, the lack of more detailed clinical data may have caused unmeasured confounding, despite all available potential confounders were considered to adjust for differences in characteristics of patients.

## Conclusions

This research offers a portrait of the “real world” of clinical practice concerning patients after an acute myocardial infarction. We revealed the association between IH-CR and clinical outcomes among subgroups of AMI patients. IH-CR participation was strongly associated to a significant improvement of adherence to evidence-based therapies both at 6- and 12-month follow-up among AMI patients who did not undergo PCI during the index hospitalization. Moreover, participation in IH-CR program was related to significant risk reduction of all-cause mortality, hospital readmission due to cardiovascular and cerebrovascular events, and admission to the emergency department during 3-year follow-up period among NSTEMI patients who did not undergo PCI.

These findings highlight the benefits of IH-CR, even in an unfavourable context due to the smallness of referral of IH-CR patients and support the clinical practise guidelines that consider cardiac rehabilitation an integral part in the treatment of coronary artery disease. AMI patients should be referred to IH-CR program, as soon as possible after the acute event, especially for those who did not received PCI during the hospitalization, and for this reason probably will not be able to benefit from a dedicated care pathway.

However, underutilization of CR is an established worldwide issue, despite its known health benefits. Referral rates urgently need improvement, and national target CR quality improvement interventions should be supported.

## Supplementary Information

**Additional file 1: Table S1.** Data sources.

**Additional file 2: Table S2.** Algorithm for selection of the cohort.

**Additional file 3: Table S3.** Selection of comorbidities from hospital discharge records.

## Data Availability

The datasets analyzed during the current study are not publicly available due to privacy issues but are available from the corresponding author on reasonable request.

## Abbreviations

CR: Cardiac rehabilitations
AMI: Acute myocardial infarction
IH-CR: In-hospital cardiac rehabilitation
STEMI: ST-elevation AMI
NSTEMI: Non-ST-elevation AMI
PCI: Percutaneous coronary intervention
MACCE: Major adverse cardiovascular and cerebrovascular events
ED: Emergency department
ACEIs: Angiotensin converting enzyme inhibitors
ARBs: Angiotensin receptor blockers
EB: Evidence-based
HIS: Hospital Information Systems
RAD-R: Regional Admission and Discharge Rehabilitation Information System
HEIS: Healthcare Emergency Information System
MIS: Mortality Information System
MPR: Medication possession ratio
DDDs: Defined daily doses
IQR: Interquartile range
OR: Odds ratio
95%CI: 95% Confidence intervals
IRR: Incidence rate ratio
