# Supplementary material for: The impact of in-hospital cardiac rehabilitation program on medication adherence and clinical outcomes in patients with acute myocardial infarction in the Lazio region of Italy
Source: BMC Cardiovasc Disord. 2021 Sep 27;21:466. doi: 10.1186/s12872-021-02261-6 (PMC8474767; doi:10.1186/s12872-021-02261-6)
Supplement: Supplementary file 1 — Additional file 1: Table S1. Data sources. [file 12872_2021_2261_MOESM1_ESM.docx]

**Table S1.**

**Data sources**

***Hospital Information System (HIS)***

The HIS is an integrated information system designed to collect clinical and administrative information regarding hospital admissions for each patient discharged from public and private hospitals of the Lazio region. The HIS includes patients’ characteristics (single anonymous identifier, gender, date and place of birth, and place of residence); admission and discharge dates; discharge diagnoses (up to 6); procedure codes (up to 6) according to the International Classification of Disease, Ninth Revision, Clinical Modification (ICD-9-CM); hospital admission and discharge ward and a regional code that corresponds to the admitting facility.

***Regional Admission and Discharge Rehabilitation Information System (RAD-R)***

The RAD-R, active since 1^st^ May 2005, was established to adequately describe the patients who access an intensive post-acute rehabilitation pathway across Lazio Region. This information system is open to all specialized facilities in the Lazio Region which have hospital wards identified by the following disciplinary codes: 28 (spinal unit), 56 (functional recovery and rehabilitation), and 75 (neurorehabilitation). RAD-R is an extension of the flow of the hospital information system (HIS) which, since 1994, through the hospital discharge form (SDO), has been collecting and managing the analytical data of all hospital admissions (in acute and post-acute) that occur every year in the Region's Hospitalization and Care Institutes.

***Healthcare Emergency Information System (HEIS)***

The HEIS includes all visits occurred in emergency departments (ED) of the Lazio region and collects patient demographic characteristics, admission information, visit and discharge dates and hours, ICD-9-CM diagnosis at discharge, reported symptoms on arrival, status at discharge (e.g., dead, hospitalized, or discharged at home) and triage score. Triage score in Italy goes from white (comparable with level 5 in the Emergency Severity Index [23]) to red (comparable to level 1).

***Drug claims registry (Pharma)***

Information on drugs reimbursed by the national healthcare system and dispensed by public and private pharmacies or by hospital pharmacies at discharge is available from the Regional Drug Dispense Registry. The data available on each prescription includes patient's identification number, prescribing physician's number, Anatomical-Therapeutic-Chemical (ATC) code of the drug purchased, number of packs, number of units per pack, dosage, unit cost per pack and prescription date.

***Mortality Information System (MIS)***

The MIS includes the patients’ demographic characteristics (the patient’s identifier, age, gender, place and date of birth, residence, marital status, and occupation), as well as the date, place, and cause of death (codified by ICD-9 codes).
