# Supplementary material for: The impact of in-hospital cardiac rehabilitation program on medication adherence and clinical outcomes in patients with acute myocardial infarction in the Lazio region of Italy
Source: BMC Cardiovasc Disord. 2021 Sep 27;21:466. doi: 10.1186/s12872-021-02261-6 (PMC8474767; doi:10.1186/s12872-021-02261-6)
Supplement: Supplementary file 2 — Additional file 2: Table S2. Algorithm for selection of the cohort. [file 12872_2021_2261_MOESM2_ESM.docx]

**Table S2.**

**Algorithm for selection of the cohort**

ICD-9-CM codes for the identification of AMI cases. AMI was defined either as a primary diagnosis of an acute myocardial infarction (ICD-9-CM 410.xx) or as a secondary diagnosis of AMI associated with one of the following conditions as the primary diagnosis:

**ICD-9-CM code Condition**

411 Other acute and subacute forms of ischemic heart disease

413 Angina pectoris

414 Other forms of chronic ischemic heart disease

423.0 Hemopericardium

426 Conduction disorders

427 Cardiac arrhythmias, excluding 427.5 Cardiac arrest

428 Heart failure

429.5 Rupture of chordae tendineae

429.6 Rupture of papillary muscle

429.71 Acquired cardiac septal defect

429.79 Certain sequelae of myocardial infarction not elsewhere classified

429.81 Other disorders of papillary muscle

518.4 Acute edema of lung, unspecified

518.81 Acute respiratory failure

780.01 Coma

780.2 Syncope and collapse

785.51 Cardiogenic shock

799.1 Respiratory arrest

997.02 Iatrogenic cerebrovascular infarction or haemorrhage

998.2 Accidental puncture or laceration during a procedure
