# Supplementary material for: The impact of in-hospital cardiac rehabilitation program on medication adherence and clinical outcomes in patients with acute myocardial infarction in the Lazio region of Italy
Source: BMC Cardiovasc Disord. 2021 Sep 27;21:466. doi: 10.1186/s12872-021-02261-6 (PMC8474767; doi:10.1186/s12872-021-02261-6)
Supplement: Supplementary file 3 — Additional file 3: Table S3. Selection of comorbidities from hospital discharge records. [file 12872_2021_2261_MOESM3_ESM.docx]

**Table S3.**

**Selection of comorbidities from hospital discharge records**

| **Condition** | **ICD-9-CM codes** | | |
| --- | --- | --- | --- |
|  | **Index admission** | **Admissions during the 2 years prior to index admission** | |
| Malignant neoplasm | 140.0–208.9, V10 | 140.0–208.9, V10 |  |
| Diabetes |  | 250.0-250.9 | |
| Disorders of lipoid metabolism/obesity | 278.0 | 272, 278.0 | |
| Haematological diseases | 280-284, 285.0, 285.2, 285.8, 285.9, 286-289 | 280-284, 285.0, 285.2, 285.8, 285.9, 286-289 | |
| Hypertension |  | 401-405 | |
| Heart failure | 428, 402.01, 402.11, 402.91 | 428, 402.01, 402.11, 402.91 | |
| Other cardiac diseases | 393-398, 421, 422, 425, 429, 745, V15.1, V42.2, V43.2, V43.3, V45.0 | 391, 393-398, 421, 422, 425, 429, 745, V15.1, V42.2, V43.2, V43.3, V45.0 | |
| Conduction disorders / cardiac arrhythmias | 426, 427 | 426, 427 | |
| Cerebrovascular disease | 433, 437, 438 | 430-432, 433, 434, 436, 437, 438 | |
| Diseases of arteries, arterioles, and capillaries | 440-448 (excl. 441.1, 441.3, 441.5, 441.6, 444), 557.1 | 440-448, 557 | |
| Chronic obstructive pulmonary disease (COPD) | 491-492, 494, 496 | 491-492, 494, 496 | |
| Chronic nephropathies | 582-583, 585-588 | 582-583, 585-588 | |
| Chronic liver, pancreas, and digestive diseases | 571-572, 577.1-577.9, 555, 556 | 571-572, 577.1-577.9, 555, 556 | |
| Cerebrovascular revascularization | 00.61, 00.62, 38.01, 38.02, 38.11, 38.12, 38.31, 38.32 | 00.61, 00.62, 38.01, 38.02, 38.11, 38.12, 38.31, 38.32 | |
| Other operations on heart and pericardium | 35, 37.0, 37.1, 37.3, 37.4, 37.5, 37.6, 37.9 | 35, 37.0, 37.1, 37.3, 37.4, 37.5, 37.6, 37.9 | |
| Other operations on vessels | 38-39.5 (excl. 38.01, 38.02, 38.5, 38.11, 38.12, 38.31, 38.32) | 38-39.5 (excl. 38.01, 38.02, 38.5, 38.11, 38.12, 38.31, 38.32) | |
